# Effect of comprehensive nursing intervention for congenital heart disease in children: A meta-analysis

**DOI:** 10.1097/MD.0000000000031184

**Published:** 2022-10-14

**Authors:** Xueying Ding, Jiaxuan Wen, Xinxin Yue, Yudan Zhao, Cuiping Qi, Di Wang, Xiuhong Wei

**Affiliations:** a School of Nursing, Weifang Medical University, Weifang , Shandong province, China; b Department of Cardiology, Affiliated Medical College of Weifang Medical College, Weifang, Shandong province, China.

**Keywords:** children, congenital heart disease, nursing Intervention

## Abstract

This meta-analysis aimed to assess the impact of nursing interventions (e.g., educational and empathic interviewing, motor exercise, therapeutic play interventions) on the perioperative outcome of children with congenital heart disease (CHD).

**Methods:**

We searched PubMed, Embase, Web of Science, Scopus, Cochrane, EBSCO, The Chinese National Knowledge Infrastructure, Wan Fang Data and the VIP Chinese Journal Service platform from the date of database creation to August 2021. Our study adhered to the recommendations of the Cochrane Handbook and the Preferred Reporting Items for Systematic Reviews and Meta-Analyses (PRISMA) statement. RevMan 5.4 and Stata 16.0 were used to complete the meta-analysis.

**Results:**

This meta-analysis showed that comprehensive nursing intervention reduced both the length of hospital stay (weighted mean difference [WMD] = −1.982, 95%CI [−2.329, −1.634], *P* < .001) and the related risk of post-operative complications [OR = 0.345, 95%CI (0.225, 0.528), *P* < .001]. In addition, nursing intervention increased parental satisfaction with the care provided [OR = 0.308, 95%CI (1.923, 6.863), *P* < .001]. Nursing interventions have also had a positive impact in reducing preoperative anxiety [WMD = −6.721, 95% CI (−7.194, −6.249), *P *< .001] and postoperative pain [WMD = −7.103, 95% CI (−7.103, −7.663), *P *< .001] in children.

**Conclusions:**

This meta-analysis confirms the beneficial effects of comprehensive nursing interventions in terms of reduced complication rates and shorter hospital stays. The effectiveness of comprehensive nursing in reducing anxiety and pain levels was also demonstrated. The findings support the implementation of comprehensive care interventions in the perioperative period for children with CHD to improve clinical outcomes.

## 1. Introduction

Congenital heart disease (CHD) is a significant concern facing pediatric health care with a significant impact on infant morbidity and mortality worldwide.^[1]^ The prevalence of CHD is 0.8% to 1.2% of all live births, and recent studies suggest that the prevalence may even be higher than previously reported.^[2]^ CHD not only poses a serious threat to children’s lives and health, but also imposes a heavy burden on society and families, as many children with CHD require one or several high-risk invasive procedures, such as cardiac surgery and/or interventional cardiac catheterization.^[3]^ Advances in medical technology have improved short- and long-term survival rates in children with CHD, but at the same time, these children continue to face complications related to surgical anatomy and physiology.^[4]^ Therefore, it is crucial to take the right approach during perioperative care in order to avoid possible complications for patients throughout their recovery.^[5]^

As a result of in comprehensive nursing interventions, there has been a high survival rate for children with CHD in most cases.^[6]^ Comprehensive nursing interventions (e.g., deep breathing exercises, inspiratory muscle training, exercise training, etc.) mitigate the effects of postoperative anesthesia by exercising the child’s cardiopulmonary and musculoskeletal systems.^[7]^ The application of interventions such as guided imagery, music therapy, and therapeutic play can reduce preoperative anxiety and postoperative pain in children.^[8]^ These interventions are often aimed at preventing or reducing postoperative complications, particularly postoperative pulmonary complications, and accelerating the child’s postoperative recovery.^[9]^

Nurses are in close contact with children during the perioperative period to examine and identify risk factors for surgery, assess children’s needs and understand children’s vulnerabilities.^[10]^ While comprehensive nursing interventions are managed or implemented by nurses. Conventional postoperative care is based on the clinical symptoms of the child and does not meet the physiological and psychological needs of the child. It is limited to informing the child of the appropriate knowledge and lacks individualized education and non-pharmacological interventions. Differently, comprehensive care interventions through health education, psychological interventions, motor training and play therapy may improve health outcomes in children with CHD in the perioperative period.^[11]^ No final conclusions have been reached regarding the use of integrated care interventions. More research is needed, particularly to examine the potential role of integrated care interventions for children with CHD. Therefore, the purpose of this meta-analysis was to assess the effectiveness of comprehensive nursing interventions (educational and empathic interviewing, motor training, and therapeutic play interventions) in reducing postoperative complications, length of stay, and relieving pain and anxiety in children.

## 2. Methods

### 2.1. Literature search

The research team conducted a meta-analysis following the Preferred Reporting Items for Systematic Evaluation and Meta-Analysis (PRISMA) guidelines. A comprehensive literature review was conducted independently by two researchers. The reviewed databases included PubMed, Embase, Web of Science, Scopus, Cochrane, EBSCO, the Chinese National Knowledge Infrastructure, Wan Fang Data, and the VIP Chinese Journal Service platform, which were systematically searched to identify relevant articles up to August 1, 2021. This article reports the results of a literature search and does not involve any animal, cell or human experimental research. This study did not require ethics approval in China.

Our search strategy encompasses a combination of the following keywords: “Nursing” or “Nursings” combined with “Heart Defects, Congenital” or “Defect, Congenital Heart” or “Abnormality, Heart” or “Heart Abnormality” or “Congenital Heart Defect” or “Malformation of Heart” or “Malformation of Hearts” and “Child or “Children”. This study combines free words with subject words. In addition, references are retrospectively included to supplement the acquisition of relevant literature.

### 2.2. Eligibility criteria

#### 2.2.1. Types of participant

Children with CHD tested and diagnosed by electrocardiogram or X-ray were used as study subjects.

#### 2.2.2. Intervention measures

Experimental group: The experimental group received nursing interventions (educational and empathic interviewing, motor exercise, therapeutic play interventions) based on the control group.

#### 2.2.3. Outcome

The primary outcomes were complications: Mainly postoperative complications such as arrhythmia, pulmonary infection, length of stay (from the end of surgery to discharge), and satisfaction (satisfaction of family members with nursing services).

The secondary outcomes: Anxious (defined as children exhibiting negative emotional behaviors such as crying, verbal protest, decreased communication and activity, and withdrawal from interactions with medical professionals). Pain (is defined as an unpleasant sensory and emotional experience caused by tissue damage, and is a protective defense response of the body to harmful stimuli).

### 2.3. Exclusion criteria

1. Children with other systemic diseases.
2. Parents disagree with the test method.
3. Non-randomized controlled trials (review of the literature, duplicate published clinical trial literature, and animal trial research literature).
4. The literature on data reporting is incomplete.
5. Studies with insufficient data to allow interpretation of results.

### 2.4. Literature quality assessment and data extraction

#### 2.4.1. Data extraction and management

Literature screening was performed independently by two researchers. The main computer database was searched to read the title and abstracts with the help of literature management software, to screen and duplicate the literature, identify and select literature that did not meet the criteria, and finally read the full text and select the standard literature. If disagreements arise during the literature selection process, consultation with an expert or discussion with a third-party investigator is recommended. If multiple papers are published from the same study, we will select the paper with the most complete experimental data and the one that best meets the inclusion criteria. All selected literature meeting the inclusion criteria was classified and analyzed through office software: basic literature information (title, author, year of publication), general information (patient age, gender, number of cases, etc.), experimental design (intervention) and outcome index. If the units of the outcome indicators in the literature were inconsistent, the units were uniformly transformed and then subjected to subsequent data processing. If literature data is lost, authors contact the original author by email or phone to obtain the relevant information needed.

#### 2.4.2. Literature quality assessment

The assessment of the risk of bias of the selected literature was performed according to the methods recommended in the Cochrane Handbook for the Systematic Evaluation of Interventions 5.1.0, and the results were reported. Assessment items include random grouping methods, allocation of hidden scheme design, use of blind methods, reporting of results data, whether there is any selective reporting of research results, other sources of bias, etc. The results are as follows: “Yes” indicates correct methodology or complete data, indicating a low risk of bias; “unclear” indicates a medium risk of bias; and “No” indicates incorrect methodology or incomplete data, indicating a high risk of bias. Finally, the results were entered into the Revman 5.4 software and risk of bias assessment plots were exported.

### 2.5. Statistical analysis

All analyses were performed using STATA, version 16.0 (Stata Corporation, College Station, TX). Statistics for count data are expressed as the dominance ratio (OR), while those for measurement data are expressed as the weighted mean difference (WMD). The effect sizes for both count and measurement data are expressed as 95% confidence intervals (CI). For the heterogeneity test, when the statistical data *P* > .1 and I^2 ^< 50%, the high homogeneity of the research results can be considered, indicating that there is no statistical difference between the included data, so a fixed-effect model is used. When the statistics *P* ≤ .1 and I^2^ ≥ 50%, it can be assumed that there may be heterogeneity among the study results, which indicates the use of a random-effects model and the consideration of factors of heterogeneity. If a study differs significantly from all the other included studies in terms of methodology or findings, then a sensitivity analysis is performed to exclude these studies from the meta-analysis. Publication bias was assessed using Egger’s test. Unless otherwise specified, *P *< .05 was judged to be statistically significant.

## 3. Results

### 3.1. Search results

**Figure 1.**
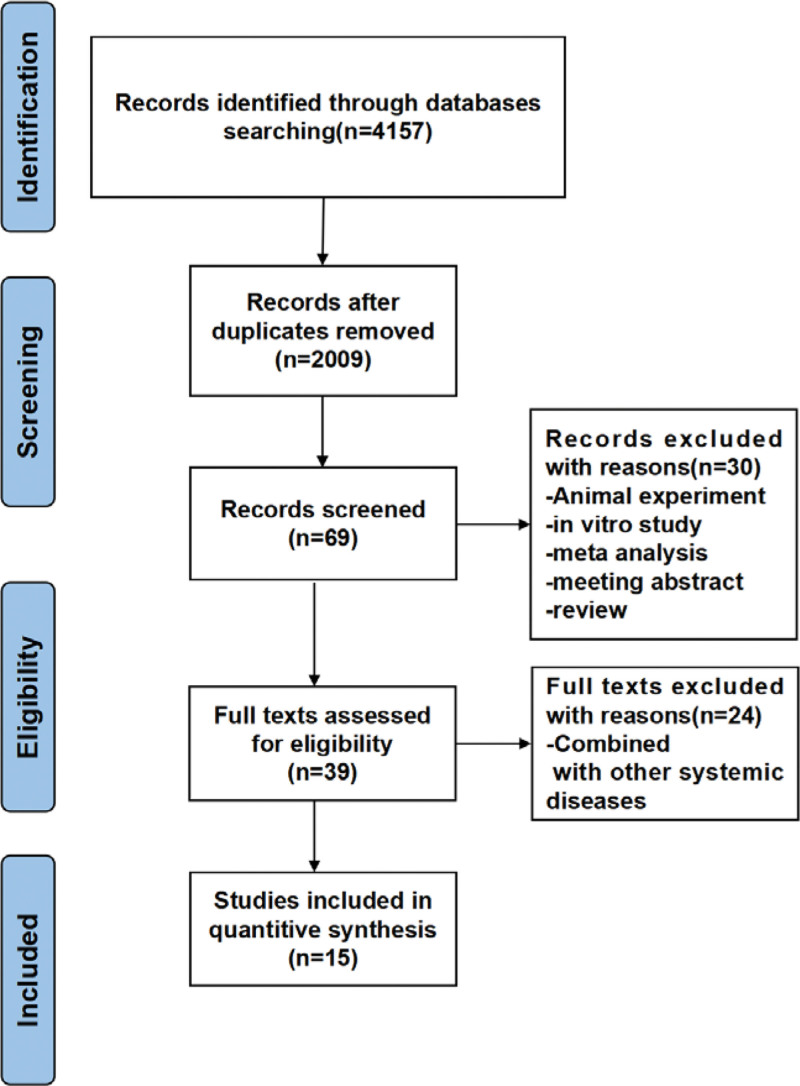
Flow chart of the study selection process.

Supplemental Digital Content 1, http://links.lww.com/MD/H649 shows the search strategy in detail. Figure 1 shows the PRISMA flow chart for literature selection and the PRISMA checklist is shown in the Supplemental Digital Content 2, http://links.lww.com/MD/H650 Table. A total of 4157 studies were found in the nine databases (PubMed = 875, Embase = 888, Web of science = 107, Scopus = 462, Cochrane = 259, EBSCO = 31, CNKI = 603, Wan Fang Data = 295 and VIP Chinese Journal Service platform = 637). No records from other sources were obtained through alternative means. After removing duplicates, 2009 records were retained; However, after reading the titles and abstracts, a further 1940 records were excluded as they were not found to be in accordance with the inclusion criteria. Sixty-nine full-text articles were read in detail to determine eligibility, of which a further 46 articles were excluded for various reasons. Finally, a total of 15 studies were included in this meta-analysis.

### 3.2. Study characteristics and quality assessment

The 15 papers included in this study were all randomized controlled trials, encompassing 10 Chinese language papers, and 5 English language literature, from a total sample size of 736 cases analyzed. The experimental and control groups contained 511 and 505 cases respectively, with a maximum sample size of 60 cases and a minimum sample size of 20 cases in each individually assessed trial. The comprehensive nursing intervention includes educational and empathic interviewing, motor exercise, and therapeutic play interventions. The outcome indicators include complication rates, length of stay, satisfaction with care, perioperative anxiety levels, and postoperative pain levels. Table 1 shows the features of articles included in this study.

**Table 1:** Characteristics of included studies.

Study	Study-type	Number of cases (test/control)	Intervention group (sample) and control group (sample)	Outcomes
Silvio 2017 (Italy)	Randomized clinical trial	57/56	Exp: Educational/informative interview (nurse visit structured with the 14 needs of Henderson, explaining the preparation for the surgery, the operating room and the perioperative process. The visit was 24–48h before the visit).	STAI
			Con: No intervention	NRS
Chen 2020 (China)	Randomized clinical trial	37/37	Exp: Formal preoperative preparation (the child life preparation program). Empathic interview (A nurse trained in communication skills does a 15 min interview following a patient-centered approach addressing questions and concerns.)	PC
			Con: post-admission nurse instruction and exercise booklet	
Feng 2016 (China)	Randomized clinical trial	31/31	Exp1: Educational/informative interview (the day before surgery. The interview included asking the patient about surgery concerns, postoperative process, education about the operating room, surgery, postoperative care)	PC
			Exp2: Multi-disciplinary training package including relaxation, respiratory training, and breathing exercises for 1 wk	LOS
			Con: Usual hospital care (oral education about deep breathing, cough and ambulation	STAI
Li 2020 (China)	Randomized clinical trial	49/48	Exp1: Gentle exercise program, stress management and relaxation, for 2 wk	PC
			Exp2: Therapeutic play intervention including tour visit, doll demonstration, and return demonstration for 1 h	LOS
			Con: Usual care	
Liao2019 (China)	Randomized clinical trial	31/31	Exp: Daily 30-min sessions of inspiratory muscle training with resistance,6 sessions/wk for 2 to 4 wk	PC
			Con: Usual care	
Luo 2020 (China)	Randomized clinical trial	20/20	Exp: Pre-operative pulmonary rehabilitation exercises twice and harmonica playing twice a wk	PC
			Con: No intervention	
Ran 2019 (USA)	Randomized clinical trial	39/39	Exp: Interview, pain relief booklet, and routine education, including booklet and video, in 1 session	PC
			Con: Routine education, including booklet and video	Nursing satisfaction
Shao2021 (China)	Randomized clinical trial	42/40	Exp: Pain relief booklet with advice to read it preoperatively, and routine education, including booklet and video, in1 session.	PC
			Con: Routine education, including booklet and video, provided in 1 session	Nursing satisfaction
Tang 2015 (China)	Randomized clinical trial	40/40	Exp1: Therapeutic play using a puppet show pertaining to the child’s operation and including a sequence of events from admission to discharge in the playroom	PC
			Exp2: Daily 30-min sessions of inspiratory muscle training with resistance,6 sessions/wk for 2 to 4 wk.	LOS
			Con: Usual care	STAI
Yang 2021 (China)	Randomized clinical trial	42/40	Exp1: Standardized preoperative preparation including a photo file, demonstration of equipment using a role modeling approach, and a tour visit for 1 h.	Nursing satisfaction
			Exp2: Instruction on deep breathing, cough and early mobilization	PC
			Con: Standard practice	LOS
Zhao 2021 (China)	Randomized clinical trial	39/39	Exp: Information modeling coping-based program: participants had a tour of the operating room, viewed a videotape, and had child-life preparation that lasted 30 min and included information on the preoperative experience and role rehearsal through photo taking and medical play	PC
				LOS
			Con: Usual care	Nursing satisfaction
Ashok 2018 (India)	Randomized clinical trial	60/60	Exp: Interview, pain relief booklet, and routine education, including booklet and video, in 1 session	Nursing satisfaction
			Con: Routine education, including booklet and video, in 1 session	STAI
				NRS
Malindi 2018 (Netherlands)	Randomized clinical trial	50/50	Exp1: Empathic interview (A nurse trained in communication skills does a 15 min interview following a children-centeredapproach addressing questions and concerns.)	Nursing satisfaction
			Exp2: Pain relief booklet with advice to read it preoperatively, and routine education, including booklet and video, in 1 session	STAI
			Con: Routine education	NRS
Feng 2020 (China)	Randomized clinical trial	31/31	Exp1: Exercise program at least 5 d preoperatively	PC
			Exp2: Children engaged in 10-min structured, fantasy play with medical materials (e.g., plastic syringes, thermometers and stethoscopes, a plastic toy operating room set, and dolls with both medical and typical clothing) while receiving verbal support from a clinical psychology graduate student	LOS
			Con: No intervention unless prescribed	STAI

Con = control group, Exp = experimental groups, LOS = length of stay, NRS = numerical rating scale, PC = postoperative complication, STAI = state-trait anxiety inventory.

**Figure 2.**
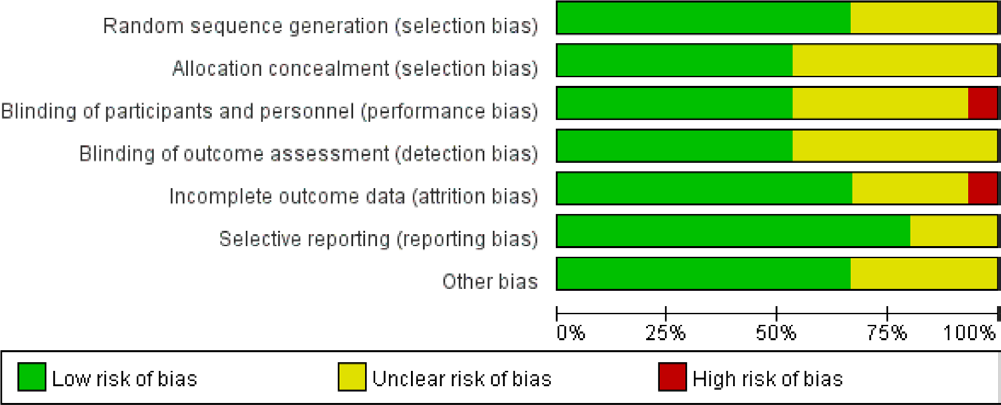
Risk of bias assessment.

**Figure 3.**
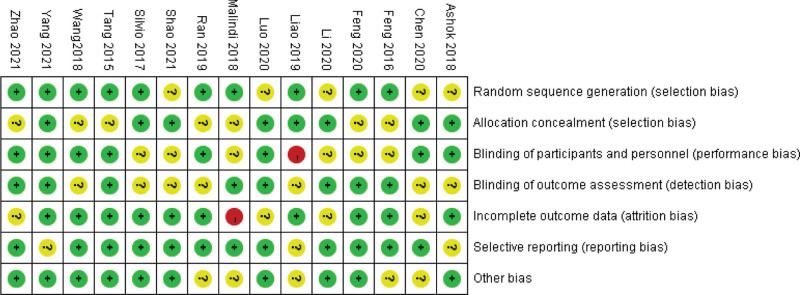
Trial quality assessment.

The risk of bias assessment is shown in detail in Figures 2 and 3. Random sequence generation and allocation concealment yielded low risks of bias between 67% and 53% of trials, respectively. In addition, blinding of participants and investigators to the assigned intervention was reported to be low in 53% of the trials, outcome assessor blinding was also reported in 53% of the trials. For each article, reasons and numbers for withdrawal and abandonment were presented. Most analyses were based on low-bias analyses.

### 3.3. Meta analysis results

#### 3.3.1. Incidence of complications

**Figure 4.**
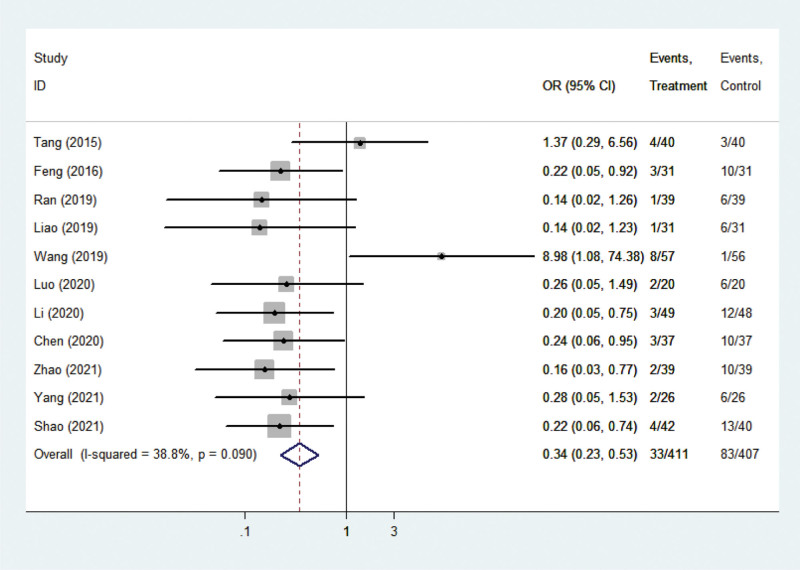
Forest plots showing the effects of nursing intervention on complications.

Eleven studies^[12–22]^ reported results regarding the incidence of postoperative adverse reactions. The studies’ statistical heterogeneity among the literature was small (*P* = .090, I^2^ = 38.3%), so a fixed-effect model of analysis was adopted. A meta-analysis of the incidence of postoperative adverse reactions resulted in a collective OR of 0.345, with a 95% CI of (0.225, 0.528), *P* < .001 (Fig. 4), indicating significant differences between comprehensive nursing intervention versus control with respect to this outcome. The incidence of adverse events in the nursing comprehensive intervention group was significantly lower than in the routine nursing group.

#### 3.3.2. Length of stay

**Figure 5.**
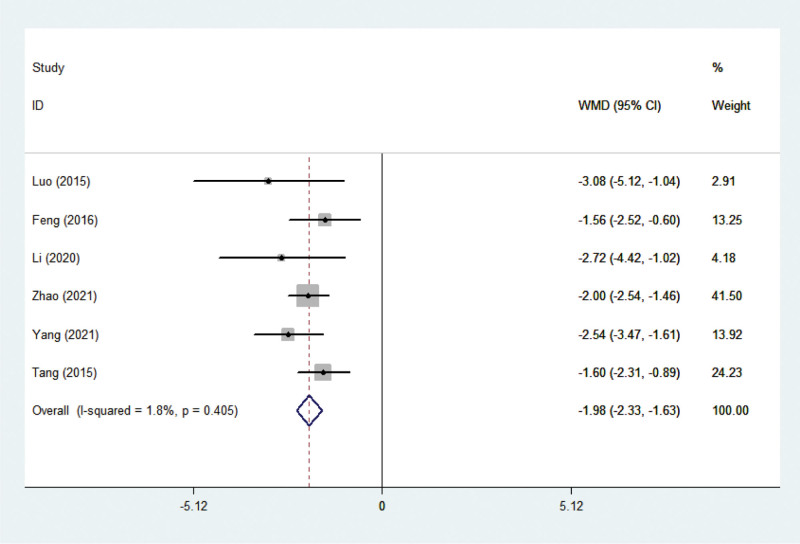
Forest plots showing the effects of nursing intervention on length of stay.

Six studies^[12,13,17,18,20,21]^ reported results on the of length of stay. There was no statistical heterogeneity among the studies (*P* = .405, I^2^ = 1.8%), and a fixed-effect model was adopted accordingly. A meta-analysis of the length of stay resulted in a collective WMD of −1.982, with a 95% CI of (−2.329, −1.634), *P *< .001 (Fig. 5), indicating significant differences between comprehensive nursing intervention versus control (routine nursing) for this outcome. The length of stay in the comprehensive nursing intervention group was significantly lower than that in the routine nursing group.

#### 3.3.3. Nursing satisfaction

**Figure 6.**
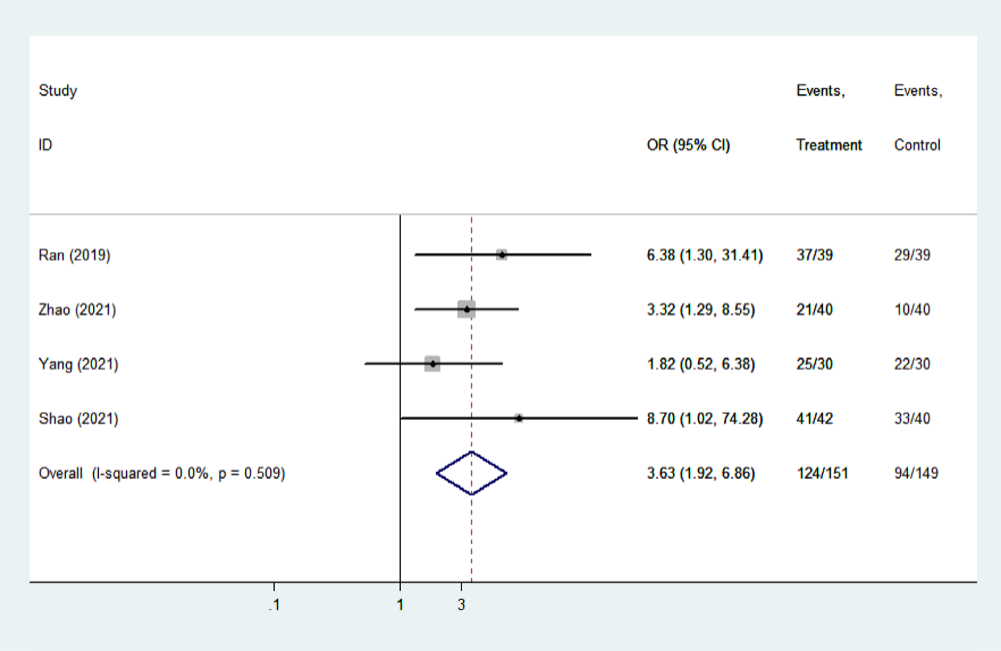
Forest plots showing the effects of nursing intervention on satisfaction.

Four studies^[14,20–23]^ reported results on nursing satisfaction. There was no statistical heterogeneity among the studies (*P* = .509, I^2^ = 0.0%), so the fixed-effect model was again adopted. A meta-analysis of nursing satisfaction resulted in a collective OR of 0.308, with a 95% CI of (1.923, 6.863), *P *< .001 (Fig. 6), indicating significant differences between comprehensive nursing intervention versus control (routine nursing) for this outcome. The satisfaction in the comprehensive nursing intervention group was significantly higher than that in the routine nursing group.

#### 3.3.4. Anxious level

**Figure 7.**
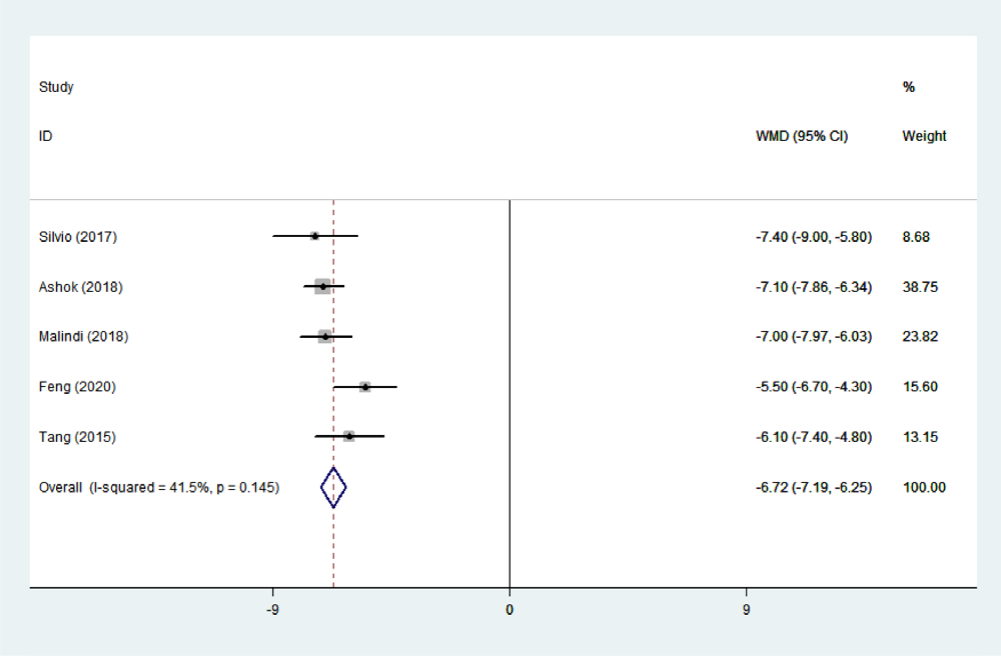
Forest plots showing the effects of nursing intervention on anxiety level.

Five studies^[11–13,23,24]^ reported the results of anxiety in children. These studies display no statistical heterogeneity in the literature (*P* = .530, I^2^ = 41.5%), and the fixed-effect model was adopted. A meta-analysis of anxious children resulted in a collective WMD of −6.721, with a 95% CI of (−7.194, −6.249), *P *< .001 (Fig. 7), indicating significant differences between comprehensive nursing intervention versus control (routine nursing) for this outcome. The incidence of anxiety level was significantly lower in the comprehensive nursing intervention group than in the routine nursing group.

#### 3.3.5. Pain level

**Figure 8.**
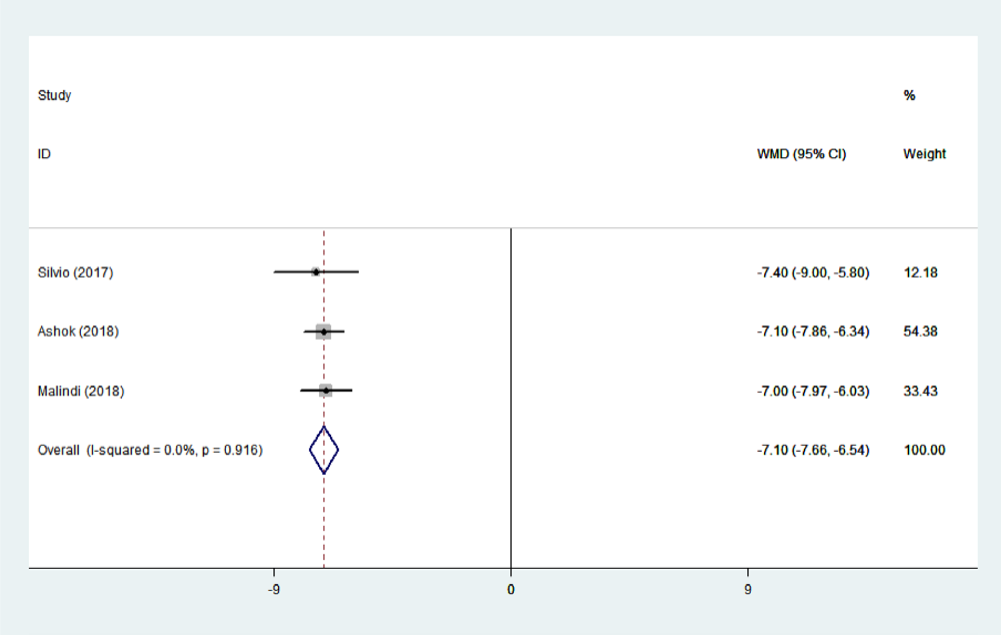
Forest plots showing the effects of nursing intervention on pain level.

Three studies^[11,23,24]^ reported results on pain level. These studies have no statistical heterogeneity in the literature (*P* = .145, I^2^ = 0.0%), and the fixed-effect model was adopted. A meta-analysis of pain levels resulted in a collective WMD of −7.103, with a 95% CI of (−7.103, −7.663), *P *< .001 (Fig. 8), indicating significant differences between nursing intervention versus control (routine nursing). The pain level was significantly lower in the comprehensive nursing intervention group than in the routine nursing group.

#### 3.3.6. Publication bias

**Figure 9.**
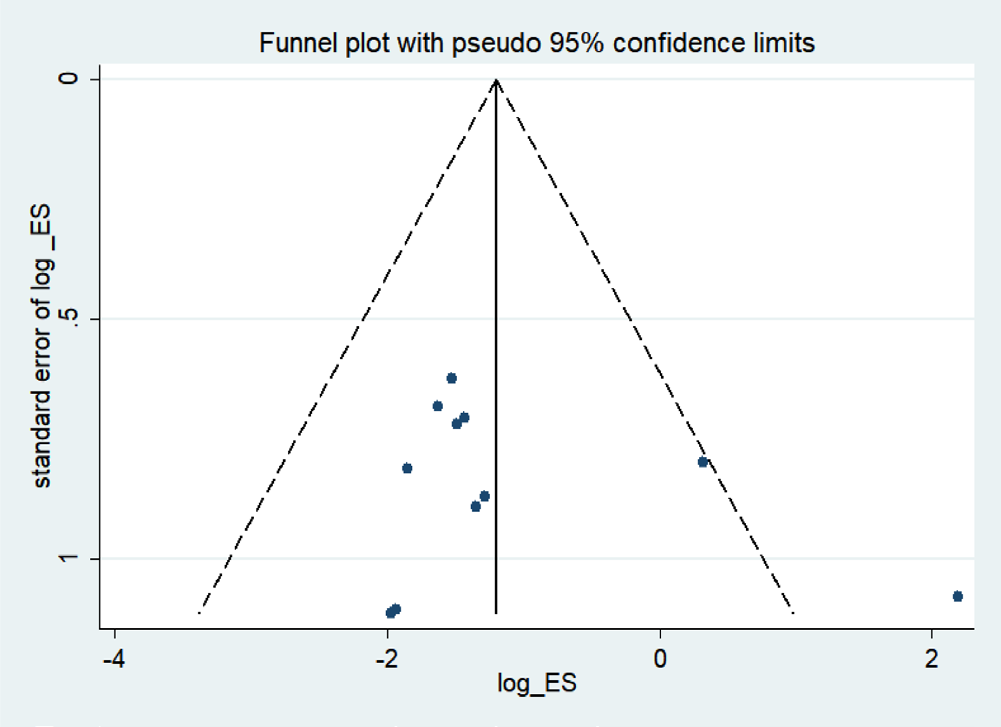
Funnel plot of complications.

The primary outcome indicator complications with the most included studies was selected for bias analysis and funnel plots were drawn, as shown in Figure 9. It can be seen from the figure that the dispersion points of each study fall within the range of the funnel plot. Meanwhile, Egger’s test for quantitative detection of publication bias showed that the incidence of complications was *P *= .336. Both methods suggested that there was a possibility of no publication bias in the study results of complication rate.

## 4. Discussion

There is compelling evidence that comprehensive nursing interventions are critical in children with CHD. In this report, we summarize for the first time the outcomes and efficacy of comprehensive nursing interventions applied to CHD in children using a meta-analysis. We found significant differences in complication rates (e.g., arrhythmias and pulmonary infections) in pediatric patients with comprehensive care interventions compared with controls, indicating that comprehensive care interventions can reduce the risk of complications. Further studies have shown that comprehensive nursing interventions can significantly reduce the length of hospital stay and alleviate anxiety and pain levels in children.

In this study, an association was found in 9 of 7 studies that assessed the relationship between comprehensive care interventions and the risk of complications. The study found that a comprehensive nursing intervention significantly reduced the rate of postoperative complications in children, which is consistent with previous meta-analysis studies.^[25]^ Alvarez study found that comprehensive care preoperative exercise interventions (such as inspiratory muscle training) significantly reduced postoperative lung infections in children.^[7]^ Rehabilitative exercise care improves autonomic function, increases maximum muscle oxygen uptake, reduces myocardial oxidative stress levels, prevents abnormal extracellular matrix collagen degradation and fibrosis, and improves ventricular remodeling and pumping function.^[26]^ Through these mechanisms, children experience significant improvements in cardiac function and a reduced incidence of arrhythmias.^[27]^ In addition, this study did not take into account the possible unobserved or unreported benefits of avoiding postoperative complications, for example, avoiding patient discomfort and the risks and costs of treatments. Further research into nursing interventions to reduce postoperative complications in children with CHD is therefore necessary.

We studied the effect of a comprehensive nursing intervention on length of stay. The comprehensive nursing intervention was able to prevent complications and thus reduce the length of stay in the pediatric ICU. This is taken into account that children undergoing cardiac surgery usually require short-term mechanical ventilation and an ICU stay. The nursing staff develops interventions for complete-operative care based on certain surgical procedures, diseases, and diagnoses, involving psychological, pharmacological, and health education interventions to shorten the length of postoperative hospital stay.^[28]^ However, Middle Eastern studies suggest that the relationship between care interventions and length of stay may not exist or may not be as significant as initially thought.^[29]^ This is because potentially unobserved variables may have a more significant impact on the length of stay. This is explained by high levels of parent and child anxiety when preoperative education is given, or background and cultural factors may have an impact.^[30]^ In addition, reducing the length of hospital stays is of considerable importance to China’s public healthcare system. This is especially true since early discharge frees up beds where hospitals can meet departmental treatment time targets.

The current meta-analysis found significant differences in parental satisfaction among children who received the integrated care intervention compared to the control group, which is consistent with a previous meta-analysis study by Doupnik et al.^[31]^ Vasli showed that the mean anxiety scores of parents who participated in and maintained comprehensive care of children were much lower than those who did not.^[32]^ The possible reasons are that comprehensive care interventions provide health guidance and training to parents who can participate in other care activities, including medication taking, providing information and invasive treatment to children, reducing their anxiety.^[33]^ Thus ensuring optimal engagement levels of parents in integrated care interventions for hospitalized children and increasing parental satisfaction with care.

In our study of children with CHD, anxiety and pain levels were significantly lower in the comprehensive care intervention group than in the control group. This finding is supported by a recently published study showing that providing nursing counseling to children undergoing congenital heart surgery reduces anxiety and stress and improves mood.^[34]^ Fun videos and play interventions for children before cardiac surgery can reduce pain.^[35]^ Some studies have also found that the evidence on the effectiveness of nursing interventions on perioperative anxiety and postoperative pain in children is unclear.^[36]^ These inconsistent findings regarding perioperative anxiety and postoperative pain in children may be due to the heterogeneity of the study design (e.g., large differences in participants from different age groups and sample sizes), outcome measures, and the timing of the measured outcomes. Participants in different age groups may have different ways of expressing anxiety and pain, leading to findings that are inconsistent with those in this review. Therefore, further research in this area is necessary.

## 5. Limitations

This meta-analysis was dominated by studies published in Chinese, with fewer studies appearing in English, limited by the number of trials. However, studies that tend to be published in multiple languages may provide a broader perspective. The impact of comprehensive nursing intervention on children with CHD and the risk of various post-operative complications still require further confirmation by more prospective randomized controlled trials.

## 6. Conclusions

In conclusion, the results of the meta-analysis of comprehensive nursing interventions for the treatment of children with CHD are reliable because they suggest that comprehensive nursing interventions reduce the risk of complications and result in reduced length of stay and improved satisfaction with care. Our evidence also suggests that comprehensive nursing interventions can improve anxiety and pain levels in children.

## Author contributions

**Data curation:** Xueying Ding, Jiaxuan Wen.

**Resources:** Xinxin Yue, Yudan Zhao.

**Software:** Cuiping Qi, Di Wang.

**Supervision:** Xiuhong Wei.

## Supplementary Material



## References

[1] Yeh SJ Chen HC Lu CW. Prevalence, mortality, and the disease burden of pediatric CHD in Taiwan. Pediatr Neonatol. 2013;54:113–8. 23590956 10.1016/j.pedneo.2012.11.010

[2] Costello JP Olivieri LJ Su L. Incorporating three-dimensional printing into a simulation-based CHD and critical care training curriculum for resident physicians. Congenit Heart Dis. 2015;10:185–90. 25385353 10.1111/chd.12238

[3] Werner O El Louali F Fouilloux V. Parental anxiety before invasive cardiac procedure in children with CHD: contributing factors and consequences. Congenit Heart Dis. 2019;14:778–84. 31066183 10.1111/chd.12777

[4] Judge P Meckler Mshs G. CHD in pediatric patients: recognizing the undiagnosed and managing complications in the emergency department. Pediatr Emerg Med Pract. 2016;13:1–28. quiz 7-8. 27096879

[5] Sjostrom-Strand A Terp K. Parents’ experiences of having a baby with a congenital heart defect and the child’s heart surgery. Compr Child Adolesc Nurs. 2019;42:10–23. 28786702 10.1080/24694193.2017.1342104

[6] Saxena A. CHD in India: a status report. Indian Pediatr. 2018;55:1075–82. 30745481

[7] Álvarez-García C Yaban Z. The effects of preoperative guided imagery interventions on preoperative anxiety and postoperative pain: a meta-analysis. Complement Ther Clin Pract. 2020;38:101077. 32056813 10.1016/j.ctcp.2019.101077

[8] Gómez-Ur JL Hueso-Montoro C Urquiza-Olmo J. A randomized controlled trial of the effect of a photographic display with and without music on pre-operative anxiety. J Adv Nurs. 2016;72:1666–76. 26880679 10.1111/jan.12937

[9] Fincher W Shaw J Ramelet AS. The effectiveness of a standardised preoperative preparation in reducing child and parent anxiety: a single-blind randomised controlled trial. J Clin Nurs. 2012;21:946–55. 22300416 10.1111/j.1365-2702.2011.03973.x

[10] Marinelli V Danzi OP Mazzi MA. PREPARE: PreoPerative anxiety REduction. One-year feasibility RCT on a brief psychological intervention for pancreatic cancer patients prior to major surgery. Front Psychol. 2020;11:362. 32194490 10.3389/fpsyg.2020.00362 PMC7066303

[11] Kumar A Das S Chauhan S. Perioperative anxiety and stress in children undergoing congenital cardiac surgery and their parents: effect of brief intervention-a randomized control trial. J Cardiothorac Vasc Anesth. 2019;33:1244–50. 30243867 10.1053/j.jvca.2018.08.187

[12] Shaomei T Jieyuan L Miao C. Effect analysis of comprehensive nursing on interventional treatment of CHD in children. Modern Med Health. 2015;21:3331–2, 3.

[13] Ya F Min C. Analysis on the effect of comfort nursing in operation of CHD in children. Res Chin Foreign Women’s Health. 2016;17:92–110.

[14] Qin R Hongying Z Youchun T. Effect of humanized nursing on complications and systemic inflammatory reaction after cardiopulmonary bypass in children with CHD. Int J Nurs. 2019;03:406–9.

[15] Yanjun L. Observation on the effect of quality nursing in the operation of CHD in children. Continuing Med Educ China. 2019;11:188–91.

[16] Wang W. Rapid rehabilitation nursing in relieving unhealthy mood and improving the prognosis of children who underwent cardiac surgery. Int J Clin Exp Med. 2019;12:12356.

[17] Hua L. Study on the application effect of comprehensive nursing in children with atrial septal defect. Contemporary Nurses (last issue). 2020;27:108–9.

[18] Shan L. Effect of ICU quality nursing intervention in early postoperative ventilator treatment of children with CHD. Med Theory Pract. 2020;33:1680–1.

[19] Xiaoxia C Li Y. Effect of comprehensive nursing intervention in early postoperative ventilator treatment of children with CHD and its influence on complication rate. Mother Baby World. 2020;10:197.

[20] Juan Z Fang W. Clinical effect of individualized nursing mode in interventional treatment of CHD in children. Chin Med Clin. 2021;21:2407–8.

[21] Qing Y. Effect analysis of comfort nursing in interventional treatment of CHD in children. Chin Health Nutrition. 2021;31:147.

[22] Yaxin S Hongjuan J Jinghua C. Effect of targeted nursing intervention on improving the safety and complications of interventional therapy for CHD in children. Int J Nurs. 2021;40:1314–7.

[23] van der Mheen M van Beynum IM Dulfer K. The CHIP-Family study to improve the psychosocial wellbeing of young children with CHD and their families: design of a randomized controlled trial. BMC Pediatr. 2018;18:230. 30001701 10.1186/s12887-018-1183-y PMC6044004

[24] Simeone S Pucciarelli G Perrone M. Comparative analysis: implementation of a pre-operative educational intervention to decrease anxiety among parents of children with CHD. J Pediatr Nurs. 2017;35:144–8. 28131545 10.1016/j.pedn.2017.01.008

[25] Thambo JB. Transcatheter interventions in CHD: we must have the means to fulfil our ambitions. Arch Cardiovasc Dis. 2020;113:89–91. 31983654 10.1016/j.acvd.2019.12.001

[26] Noone C Dwyer CP Murphy J. Comparative effectiveness of physical activity interventions and anti-hypertensive pharmacological interventions in reducing blood pressure in people with hypertension: protocol for a systematic review and network meta-analysis. Syst Rev. 2018;7:128. 30131071 10.1186/s13643-018-0791-9 PMC6103808

[27] Giallauria F Acampa W Ricci F. Exercise training early after acute myocardial infarction reduces stress-induced hypoperfusion and improves left ventricular function. Eur J Nucl Med Mol Imaging. 2013;40:315–24. 23224706 10.1007/s00259-012-2302-x

[28] Faerber JA Huang J Zhang X. Identifying risk factors for complicated post-operative course in tetralogy of fallot using a machine learning approach. Front Cardiovasc Med. 2021;8:685855. 34368247 10.3389/fcvm.2021.685855 PMC8339319

[29] Snowdon D Haines TP Skinner EH. Preoperative intervention reduces postoperative pulmonary complications but not length of stay in cardiac surgical patients: a systematic review. J Physiother. 2014;60:66–77. 24952833 10.1016/j.jphys.2014.04.002

[30] Alghatani K Ammar N Rezgui A. Predicting intensive care unit length of stay and mortality using patient vital signs: machine learning model development and validation. JMIR Med Inform. 2021;9:e21347. 33949961 10.2196/21347 PMC8135024

[31] Doupnik SK Hill D Palakshappa D. Parent coping support interventions during acute pediatric hospitalizations: a meta-analysis. Pediatrics. 2017;140. 10.1542/peds.2016-4171 PMC5574731 28818837

[32] Vasli P Salsali M. Parents’ participation in taking care of hospitalized children: a concept analysis with hybrid model. Iran J Nurs Midwifery Res. 2014;19:139–44. 24834082 PMC4020022

[33] Muhly WT Wohler B Nelson MN. A qualitative assessment of factors that children, parents, and clinicians prioritize in the setting of elective anesthesia and surgery. Anesth Analg. 2021;132:1067–74. 32502137 10.1213/ANE.0000000000004936

[34] Li X Qiao XF Sun L. Application of situational adaptation training combined with childlike nursing for children undergoing tonsillectomy or adenoidectomy. Int J Pediatr Otorhinolaryngol. 2021;145:110707. 33887548 10.1016/j.ijporl.2021.110707

[35] Rantala A Pikkarainen M Miettunen J. The effectiveness of web-based mobile health interventions in paediatric outpatient surgery: a systematic review and meta-analysis of randomized controlled trials. J Adv Nurs. 2020. 10.1111/jan.14381 32281673

[36] Mathias EG Pai MS. Anxiety and pain in children undergoing surgery: a scoping review. J Perianesthesia Nurs. 2022;37:545–50. 10.1016/j.jopan.2021.10.002 35279386

