# Supplementary material for: Effect of comprehensive nursing intervention for congenital heart disease in children: A meta-analysis
Source: Medicine (Baltimore). 2022 Oct 14;101(41):e31184. doi: 10.1097/MD.0000000000031184 (PMC9575750; doi:10.1097/MD.0000000000031184)
Supplement: Supplementary file 1 [file medi-101-e31184-s001.pdf]

Supplementary material

Search strategy

CNKI: (subject = Nursing) and (subject = children) and (subject = congenital heart disease) 603

Wang Fang Date: All: (Nursing) and all: (children) and all: (congenital heart disease) and all: (randomized control) 295

VIP: ((any field = nursing and any field = children) and title or keyword = congenital heart disease) 637

PubMed: 875

#3 AND #6 AND #10

#10 "Lower Extremity"[Mesh]

#9 #7 OR #8 2893491

#8 (Children[All Fields]) OR (Child[All Fields])

#7 "Children"[Mesh]

#6 #4 OR #5 1578361

#5 (((((((Heart Defects, Congenital[All Fields) OR (Defect, Congenital Heart[All Fields)) OR (Abnormality, Heart[All Fields)) OR (Heart Abnormality[All Fields)) OR (Congenital Heart Defect[All Fields)) OR (Heart, Malformation Of[All Fields)) OR (Malformation Of Heart[All Fields)) OR (Malformation Of Hearts[All Fields)

#4 "Congenital heart disease"[Mesh]

#3 #1 OR #2 850182

#2 (Nursing[All Fields]) OR Nursings [All Fields])

#1 "Nursing"[Mesh]

Web of Science:431

#1 AND #2 AND #3

#1 TS=((Nursing) OR (Nursings)) 197892

#2 TS=((((((((Heart Defects, Congenital) OR (Defect, Congenital Heart)) OR (Abnormality, Heart)) OR (Heart Abnormality)) OR (Congenital Heart Defect)) OR (Heart, Malformation Of)) OR (Malformation Of Heart)) OR (Malformation Of Hearts) 33194

#3 TS=((Child) OR (Children)) 99214

Scopus:462

#1 AND #2 AND #3

#1 TITLE-ABS-KEY((nursing) OR (nursings)) 697598

#2 TITLE-ABS-KEY(((((((Heart Defects, Congenital) OR (Defect, Congenital Heart)) OR (Abnormality, Heart)) OR (Heart Abnormality)) OR (Congenital Heart Defect)) OR (Heart, Malformation Of)) OR (Malformation Of Heart)) OR (Malformation Of Hearts) 174091

#3 TITLE-ABS-KEY((child)OR(children)) 3317007

EBSCO:31

(S1 AND S2 AND S3)

#1 (Nursing) OR (Nursings) 369156

#2 (((((((Heart Defects, Congenital) OR (Defect, Congenital Heart)) OR (Abnormality, Heart)) OR (Heart Abnormality)) OR (Congenital Heart Defect)) OR (Heart, Malformation Of)) OR (Malformation Of Heart)) OR (Malformation Of Hearts) 6577

#3 (Child) OR (Children) 3741067

Cochrane:295

#1 AND #2 AND #3

#1 (Nursing) OR (Nursings) in All Text - (Word variations have been searched) 2870

#2 (((((((Heart Defects, Congenital) OR (Defect, Congenital Heart)) OR (Abnormality, Heart)) OR (Heart Abnormality)) OR (Congenital Heart Defect)) OR (Heart, Malformation Of)) OR (Malformation Of Heart)) OR (Malformation Of Hearts) in All Text - (Word variations have been searched) 1165

#3 (Child) OR (Children) in All Text - (Word variations have been searched) 4386

Embase:888

#1 AND #2 AND #3

#1 Nursing OR Nursings 1016338

#2 (((((((Heart Defects, Congenital) OR (Defect, Congenital Heart)) OR (Abnormality, Heart)) OR (Heart Abnormality)) OR (Congenital Heart Defect)) OR (Heart, Malformation Of)) OR (Malformation Of Heart)) OR (Malformation Of Hearts) in All Text - (Word variations have been searched) 132536

#3 Child OR Children 3631610
